# Use of intraventricular ribbon gauze to reduce particulate emboli during aortic valve replacement

**DOI:** 10.1186/1749-8090-1-42

**Published:** 2006-11-07

**Authors:** Mahmoud Loubani, Daniel Von Petius, Paul D Ridley

**Affiliations:** 1 Department of Cardiothoracic Surgery, University Hospitals of North Staffordshire, Stoke-On-Trent, UK; 2 Department of Pathology, University Hospitals of North Staffordshire, Stoke-On-Trent, UK; 3 Department of Cardiothoracic Surgery, University Hospitals of Coventry and Warwickshire NHS Trust, Walsgrave Hospital, Clifford Bridge Road, Coventry, CV2 2DX, UK

## Abstract

**Background:**

The incidence of cerebrovascular accidents following aortic valve surgery remains a devastating complication. The aim of this study was to determine the number of potential embolic material arising during aortic valve replacement and to examine the efficacy of using ribbon gauze in the left ventricle during removal of the native valve and decalcification of the aortic annulus.

**Methods:**

Ribbon gauze was inserted into the left ventricular cavity prior to aortic valve excision in an unselected, prospectively studied series of 30 patients undergoing aortic valve replacement. A further 30 lengths of ribbon gauze were soaked in the pericardiotomy blood of the same patients and all were subjected to histological analysis.

**Results:**

The median number of tissue fragments from the aortic valve replacement group was significantly higher than in the control group 5 (0–18) versus 0 (0–1) (p = 3.6 × 10^-5^). The size of tissue fragments varied between 0.1 and 9.0 mm with a mean of 0.61 ± 1.12 mm and a median of 0.2 mm. There was a significantly higher number of tissue fragments associated with patients having surgery for aortic stenosis when compared with patients who had aortic regurgitation with median of 5 (0–18) versus 0 (0–3) (p = 0.8 × 10^-3^).

**Conclusion:**

Significant capture of particulate debris by the intraventricular ribbon gauze suggests that the technique of left ventricular ribbon gauze insertion during aortic valve excision has merit.

## Background

Aortic valve replacement has been shown to be associated with postoperative cerebrovascular accidents in approximately 10% of cases in several studies [1,2], which may be related to embolic events [3,4]. This most devastating morbidity has a significant impact on survival and quality of life [5]. We have routinely employed the technique of inserting ribbon gauze into the left ventricle during excision of the native aortic valve with the aim of capturing embolic debris. In this investigation we aim to validate this technique by a prospective study of a sequential unselected series of thirty patients undergoing aortic valve surgery.

## Materials and methods

### Patient selection

Thirty consecutive patients undergoing aortic valve surgery for significant aortic valve disease were included in this study.

### Study design and surgical technique

In this prospective study 60 ribbon gauzes were either placed in the left ventricle prior to removal of the native aortic valve and decalcification of the aortic annulus during aortic valve replacement or soaked in the pericardiotomy blood. A single surgeon performed all operations (PDR). Cardiopulmonary bypass was established with standard techniques employing an ascending aortic and a two-stage venous cannula. A left ventricular vent was inserted via the right superior pulmonary vein. The heart was protected with antegrade and retrograde cold blood cardioplegia; systemic core cooling to 34°C and topical slush was placed on the heart. The operative field was flooded with CO_2 _delivered at 4 L/min. An oblique aortotomy was performed to gain access to the aortic valve. A length of ribbon gauze (1 cm × 30 cm) was soaked in saline and then inserted into the left ventricle prior to valve excision. After excision of the aortic valve using sharp dissection and thorough debridement of the aortic annulus, the ribbon gauze was carefully removed and sent in normal saline for histological analysis. Using a large syringe, the left ventricular cavity was washed with an injection of cold normal saline, which was sucked out and discarded. Aortic valve replacement with or without adjunctive procedures was then carried out.

### Assessment of ribbon gauze

The ribbons were blindly assessed by an independent pathologist (DVP). Every specimen was examined separately documenting the specimen number. The gauze within each pot was examined first, taking care to leave all excess formalin within the pot. Each gauze was examined under a 10× microscope to ensure the detection of all fragments >0.1 mm. Each fragment was measured and described regarding colour and consistency prior to placement within a sachet designed to retain small tissue particle. The remaining formalin within the pot was then strained through a funnel into the sachet to catch all the remaining tissue fragments. These fragments were described, measured and placed and processed using the normal standard processing procedures. A single Hematoxylin and Eosin stained section was cut from each specimen to allow the microscopic examination under higher magnification (100×) to describe the type and character of the tissue.

### Statistical analysis

Data are expressed as median and range as well as mean ± standard deviation where appropriate. Non parametric data in the various groups were compared using Chi-square test and value of p < 0.05 was taken as statistically significant.

## Results

### Patient outcome

The patient's preoperative demographic and clinical characteristics are included in Table 1 and operative and postoperative data are shown in Table 2. None of the patients developed a postoperative cerebrovascular event but there was one death secondary to sepsis followed by renal and respiratory failure 32 days postoperatively.

**Table 1:** Preoperative clinical characteristics

Mean Age	65.9 ± 8.9 years
Male:Female	19:11
Aortic valve disease	
Stenosis	25
Regurgitation	5
Coronary artery disease	13
Left ventricular ejection fraction	
>50%	25
30–49%	5
<30%	2
Parsonnet score	11.2 ± 7.6

**Table 2:** Intraoperative and postoperative data

Cardiopulmonary Bypass Time	140.1 ± 48.9 minutes
Aortic Cross Clamp Time	101.5 ± 33.5 minutes
Mean postopertive ITU Stay	37.6 ± 72.4 hours
Mean Postopertive Hospital Stay	10.8 ± 14.1 days
Concomitant procedure	
Coronary artery bypass grafts surgery	13
Radiofrequency ablation	4

### Histological findings

**Figure 1:**
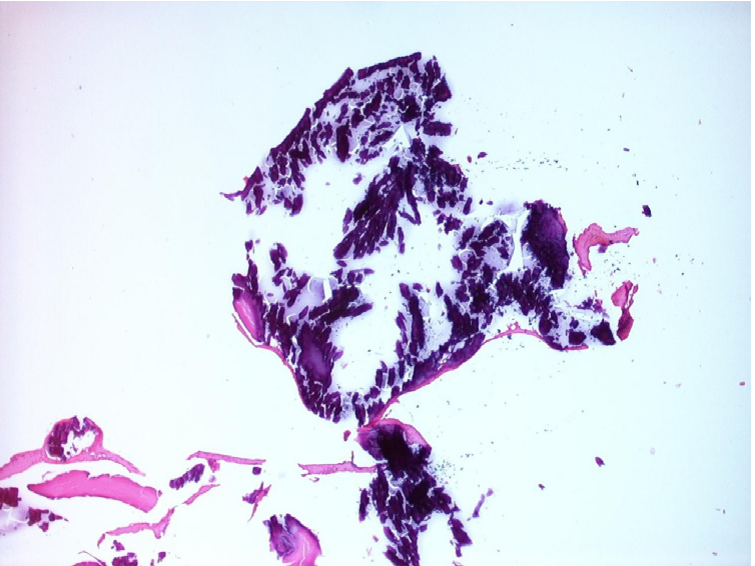
Heavy calcified tissue fragment, approximately 1.5 mm diameter (H&E, 40× magnification).

**Figure 2:**
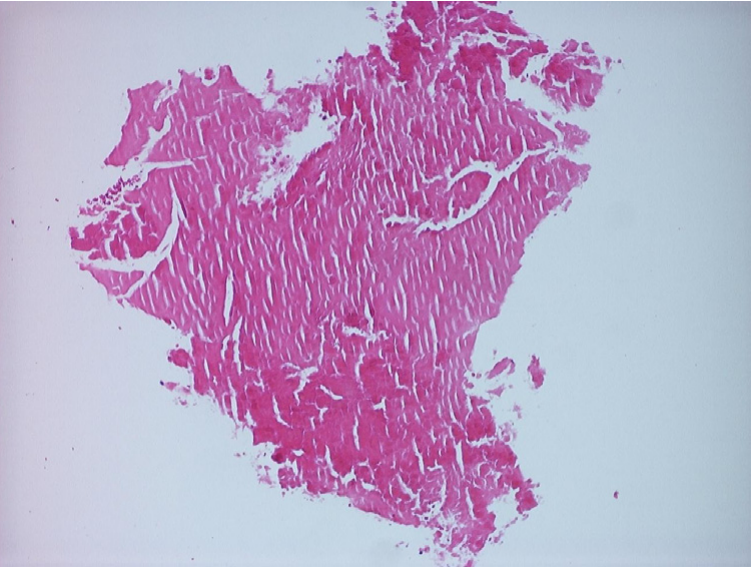
Non-calcified hyaline tissue, approximately 1 mm diameter, (H&E, 100× magnification).

Sixty pieces of ribbon gauze were examined and the mean number of embolic material from the aortic valve replacement group with a median of 5 (0–18) was significantly higher than in the control group with a median of 0 (0–1) (p = 3.6 × 10^-5^). The size of emboli varied between 0.1 and 9.0 mm with a mean of 0.61 ± 1.12 mm and median of 0.2 mm. There was a significantly higher number of debris associated with patients that had surgery for aortic stenosis with a median of 5 (range 0–18) when compared with patients that had aortic regurgitation with a median 0 (range 0–3) (p = 0.8 × 10^-3^). The debris varied from heavily calcified fragments to non-calcified tissue as seen in Figures 1 and 2.

## Discussion

Cerebrovascular accidents remain the most devastating of the nonfatal complications of open-heart surgery. Cerebral injury has been studied in greatest detail in patients undergoing coronary artery bypass graft surgery and emphasis has been placed on the association of ascending aortic atheroma and perioperative cerebral injury [6]. The incidence of cerebral injury may be even higher in the group of patients undergoing AVR [1,2,4]. This might be expected to be most at risk are those patients with severely calcified aortic valves and we found more evidence of ribbon gauze debris in those patients with aortic stenosis than in those with aortic regurgitation. Modified operative techniques have been employed to attempt to reduce particulate [7,8] and gaseous [9] emboli associated with open-heart surgery.

The logic of the use of intraventricular ribbon gauze by some cardiac surgeons during aortic valve replacement is to capture any particles that might fall into the ventricle during valve debridement. All usual methods using observant careful surgical technique to excise the valve must be employed. Excision of the aortic valve should be an unhurried process with great care being taken to avoid dislodged particulate matter falling into the left ventricle or the dependent left coronary ostium. The simple additional procedure to introduce the ribbon gauze into the ventricular cavity does not add to the time taken for the procedure and acts as an added safety net. The removal of the ribbon gauze must be documented by the theatre staff prior to valve insertion

The study population made satisfactory clinical progress and there were no cerebrovascular accidents. Our study shows significant capture of particulate debris by the intraventricular ribbon gauze compared to specimens placed in pericardiotomy blood. This occurred despite all efforts being made to carefully excise and debride the aortic valve, which suggests that the technique has merit.

## Authors' contributions

PDR and ML designed the study, carried out the surgery, collected the data and perpared the manuscript. DVP carried out all the histopathological investigations and helped in preparation of the manuscript. All authors read and approved the final manuscript.

## References

[B1] Stolz E, Gerriets T, Kluge A, Klovekorn WP, Kaps M, Bachmann G (2004). Diffusion-weighted magnetic resonance imaging and neurobiochemical markers after aortic valve replacement implications for future neuroprotective trials?. Stroke.

[B2] Wolman RL, Nussmeier NA, Aggarwal A, Kanchuger MS, Roach GW, Newman MF, Mangano CM, Marschall KE, Ley C, Boisvert DM, Ozanne GM, Herskowitz A, Graham SH, Mangano DT (1999). Multicenter Study of Perioperative Ischemia Research Group (McSPI) and the Ischemia Research Education Foundation (IREF) Investigators Cerebral injury after cardiac surgery identification of a group at extraordinary risk. Stroke.

[B3] Zimpfer D, Czerny M, Kilo J, Kasimir M-T, Madl C, Kramer L, Wieselthaler GM, Wolner E, Grimm M (2002). Cognitive deficit after aortic valve replacement. Ann Thorac Surg.

[B4] Braekken SK, Reinvang I, Russell D, Brucher R, Svennevig JL (1998). Association between intraoperative cerebral microembolic signals and postoperative neuropsychological deficit: comparison between patients with cardiac valve replacement and patients with coronary artery bypass grafting. J Neurol Neurosurg Psychiatr.

[B5] Hogue CW, Murphy SF, Schechtman KB, Dávila-Román VG (1999). Risk Factors for Early or Delayed Stroke After Cardiac Surgery. Circulation.

[B6] Mills NL, Everson CT (1991). Atherosclerosis of the ascending aorta and coronary artery bypass: pathology, clinical correlates and operative management. J Thorac Cardiovasc Surg.

[B7] Ridley PD, Hendel PN, Thomson DS (1994). Atherosclerotic ascending aorta: management during coronary artery bypass graft surgery. Asia Pacific J Thorac Cardiovasc Surg.

[B8] Banbury MK, Kouchoukos NT, Allen KB, Slaughter MS, Weissman NJ, Berry GJ, Horvath KA, the ICEM 2000 investigators (2003). Emboli capture using Embol-X intraartic filter in cardiac surgery: a multicentreed randomized trial of 1,289 patients. Ann Thorac Surg.

[B9] Webb WR, Harrison LH, Hemcke FR, Camio-Lopez A, Munfakh NA, Heck HA, Moulder PV (1997). Carbon Dioxide field flooding minimizes residual intracardiac air after open heart surgery. Ann Thorac Surg.

